# Synergistic Targeting of Innate Receptors TLR7 and NOD2 for Therapeutic Intervention in Multiple Sclerosis

**DOI:** 10.3390/ijms25137462

**Published:** 2024-07-07

**Authors:** Magdalena Dubik, Joanna Marczynska-Grzelak, Michael Zaucha Sørensen, Ruthe Storgaard Dieu, Dominika Rusin, Eydís Sigurdardóttir Schiöth, Bita Ramazani, Rouhin Belal, Bhavya Ojha, Jonathan Krieger, Dina S. Arengoth, Agnieszka Wlodarczyk, Trevor Owens, Reza Khorooshi

**Affiliations:** Department of Neurobiology Research, Institute of Molecular Medicine, University of Southern Denmark, Campusvej 55, DK-5000 Odense C, Denmark

**Keywords:** TLR7, NOD2, synergy, type I IFN, experimental autoimmune encephalomyelitis

## Abstract

Regulation of neuroinflammation is critical for maintaining central nervous system (CNS) homeostasis and holds therapeutic promise in autoimmune diseases such as multiple sclerosis (MS). Previous studies have highlighted the significance of selective innate signaling in triggering anti-inflammatory mechanisms, which play a protective role in an MS-like disease, experimental autoimmune encephalomyelitis (EAE). However, the individual intra-CNS administration of specific innate receptor ligands or agonists, such as for toll-like receptor 7 (TLR7) and nucleotide-binding oligomerization-domain-containing protein 2 (NOD2), failed to elicit the desired anti-inflammatory response in EAE. In this study, we investigated the potential synergistic effect of targeting both TLR7 and NOD2 simultaneously to prevent EAE progression. Our findings demonstrate that simultaneous intrathecal administration of NOD2- and TLR7-agonists led to synergistic induction of Type I IFN (IFN I) and effectively suppressed EAE in an IFN I-dependent manner. Suppression of EAE was correlated with a significant decrease in the infiltration of monocytes, granulocytes, and natural killer cells, reduced demyelination, and downregulation of *IL-1β*, *CCL2*, and *IFNγ* gene expression in the spinal cord. These results underscore the therapeutic promise of concurrently targeting the TLR7 and NOD2 pathways in alleviating neuroinflammation associated with MS, paving the way for novel and more efficacious treatment strategies.

## 1. Introduction

Multiple sclerosis (MS) is a chronic autoimmune disease characterized by inflammation and demyelination within the central nervous system (CNS), leading to neurological dysfunction and disability [1]. Despite advancements in understanding the pathophysiology of MS, current treatment options remain limited and often associated with significant side effects. Neuroinflammation, orchestrated by dysregulated immune responses, plays a pivotal role in MS progression and pathogenesis. Thus, modulating immune responses and restoring CNS homeostasis represent attractive therapeutic approaches for MS management. Recent studies have highlighted the significance of selective innate signaling in triggering anti-inflammatory mechanisms, including the production of IFN I, which play a protective role in the MS-like disease experimental autoimmune encephalomyelitis (EAE) [2,3]. However, the individual intra-CNS administration of specific innate receptor ligands or agonists, such as toll-like receptor 7 (TLR7) was insufficient to elicit an anti-inflammatory response in EAE [4]. Similarly, in this study, we found that the stimulation of nucleotide-binding oligomerization domain-containing protein 2 (NOD2) had no beneficial effect on EAE when given at the time of disease onset.

NOD2 is a cytosolic receptor primarily responsible for detecting bacterial peptidoglycan fragments (bacterial muramyl dipeptide, MDP), while TLR7 is an endosomal receptor that responds to viral single-stranded RNA [5,6]. Co-stimulation with NOD2 and TLR7 agonists has been reported to synergistically enhance cytokine production in peripheral blood mononuclear cells and macrophages [7,8]. Additionally, simultaneous targeting of both receptors produces super-additive effects on the development of immune responses in vivo [9].

Recent studies have demonstrated that the administration of an immunomodulatory microparticle that activates TLR9 and NOD2 signaling is protective against EAE [10,11]. To investigate the potential synergistic effect of targeting CNS innate receptors TLR7 and NOD2 in preventing EAE, we employed intrathecal administration of specific agonists for TLR7 and NOD2 in combination. Our results indicate that while individual administration of TLR7 or NOD2 agonists failed to induce an anti-inflammatory program in EAE, simultaneous activation of both receptors resulted in a synergistic induction of IFN I. Furthermore, intrathecal administration of combined NOD2 and TLR7 agonists effectively reduced infiltration of immune cells into the CNS and suppressed EAE progression in an IFN I-dependent manner.

## 2. Results

### 2.1. While Individual Intrathecal Delivery of Imiquimod or MDP Failed to Suppress EAE, Their Combination Had Disease-Modifying Activity

Previously, we reported that the stimulation of TLR7 within the CNS had no beneficial effects on EAE [4]. Similarly, in this study, stimulation of NOD2 by MDP within the CNS did not alter the progression of EAE (Supplementary Figure S1). We and others have shown that the delivery of a bispecific immunomodulatory microparticle that activates TLR9 and NOD2 signaling reduces the severity of EAE [10,11]. Together, these findings prompted us to examine the potential synergistic impact of concurrently targeting both TLR7 and NOD2 on EAE progression.

**Figure 1:**
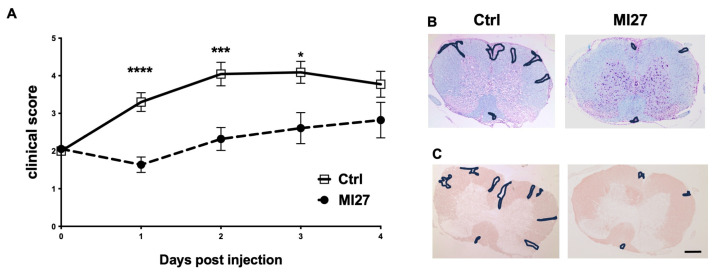
Intrathecal MI27 suppressed EAE. (**A**) C57BL/6 mice were immunized with MOGp35-55 to induce EAE, and at disease onset, they received intrathecal MI27 or PBS, and clinical signs were scored daily. At the onset of EAE (day 0), mice that received MI27 showed marked attenuation of disease compared to control mice (*n* = 15–18 in each group). The data are pooled from three independent studies. Data are presented as mean ± SEM. Results were analyzed using the two-tailed Mann–Whitney u-test. * *p* < 0.05, *** *p* < 0.001, **** *p* < 0.0001. (**B**,**C**) Micrographs of spinal cord sections from mice with EAE that received MI27 or PBS intrathecally, stained with LFB and cresyl violet and with anti-MOG antibody. (**B**) In the control group, cresyl violet staining showed cell infiltration into the parenchyma of the spinal cord with extensive loss of LFB (marked area) and (**C**) MOG staining in corresponding areas (marked area). Mice with EAE that were treated with intrathecal MI27 showed cell accumulation in the meninges and reduced loss of LFB- and MOG staining. Scale bar. 200 µm.

Following EAE induction in mice, upon the manifestation of initial symptoms (floppy tail, defined as day 0), a single intrathecal injection of combined imiquimod and MDP (hereafter called MI27) was administered. Subsequent disease progression was monitored over 4 days. Intrathecal administration of MI27 resulted in significant reduction in disease severity compared to controls. The therapeutic effects of MI27 were apparent as early as day one post-treatment and persisted through day three (Figure 1A).

As expected, histological analysis of the spinal cord white matter in control animals showed increased nuclear density, indicative of leukocyte infiltration (marked regions in Figure 1B) and reduced MOG staining indicative of demyelination (marked regions in Figure 1C). Notably, treatment with MI27 led to a decrease in immune cell infiltration, primarily confined to the extraparenchymal regions within the meninges (Figure 1B). Additionally, MI27 treatment mitigated myelin loss in the spinal cord (Figure 1C).

### 2.2. Intrathecal MI27 Induced IFN Beta Response in the CNS and Suppressed EAE in an IFN I Dependent Manner

**Figure 2:**
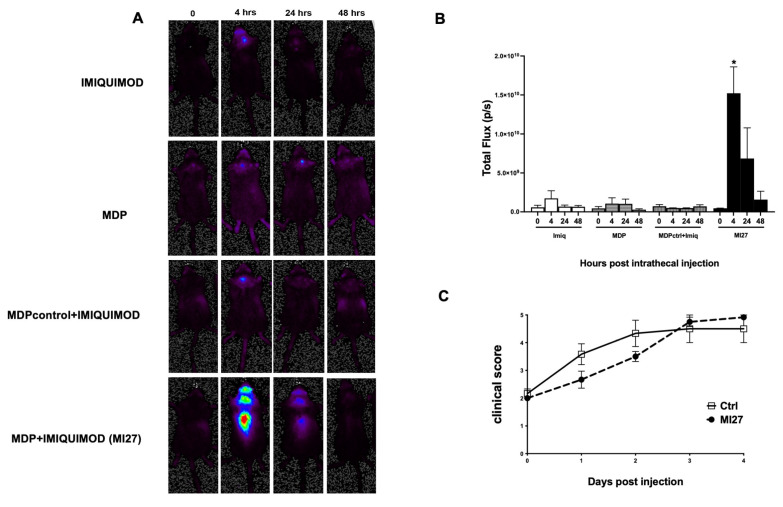
Intrathecal MI27 induced IFNβ and suppressed EAE in an IFNAR-dependent manner. (**A**,**B**) In vivo imaging of IFNβ/luciferase reporter mice that received intrathecal imiquimod, MDP, MDP control + imiquimod, or MDP control + imiquimod (MI27). The level of IFNβ was significantly increased at 4- and 24-h post-injection. *n* = 3 in each group. (**C**) IFNAR1-deficient mice were immunized with MOGp35-55 to induce EAE and at disease onset they received intrathecal MI27 or PBS, and clinical signs were scored daily. The protective effect of intrathecal MI27 was abrogated in IFNAR1-KO mice. (*n* = 6 per group). Data are presented as mean ± SEM. Results were analyzed using paired Student’s *t*-test. * *p* < 0.05.

Interferon beta (IFNβ) has demonstrated therapeutic efficacy in the treatment of MS. Given that activation of either TLR7 or NOD2 pathways can induce IFN I expression, we aimed to investigate whether intrathecal administration of MI27 induces IFNβ expression in the steady state CNS. For this purpose, we utilized mice expressing a luciferase gene under the control of the IFNβ promoter [12]. Mice received intrathecal injections of imiquimod, MDP, MDP-ctrl combined with imiquimod, or MI27. Luciferase activity indicative of IFNβ expression was measured 4, 24, and 48 h post-treatment. In vivo imaging revealed that a single intrathecal injection of MI27 significantly increased IFNβ expression within the CNS at 4 h post-injection (Figure 2A,B). In contrast, intrathecal administration of imiquimod, MDP, or MDP-ctrl + imiquimod failed to induce IFNβ expression (Figure 2B).

The robust induction of IFNβ expression observed in mice treated with MI27 strongly suggested a pivotal role for IFNAR signaling in mediating the effects of MI27. To further explore this, we investigated the potential synergistic impact of concurrently targeting both TLR7 and NOD2 on EAE progression in mice lacking IFN I signaling. IFNAR1-deficient mice were immunized to induce EAE, and upon disease onset, they received a single intrathecal injection of MI27. Disease progression was monitored for 4 days. Disease severity remained unaffected in IFNAR1-deficient mice treated with intrathecal MI27 (Figure 2C). This finding indicates that the therapeutic efficacy of MI27 is dependent on IFN I signaling pathways.

### 2.3. Intrathecal MI27 Treatment Reduced CNS Recruitment of Selected Immune Cells into the Spinal Cord

**Figure 3:**
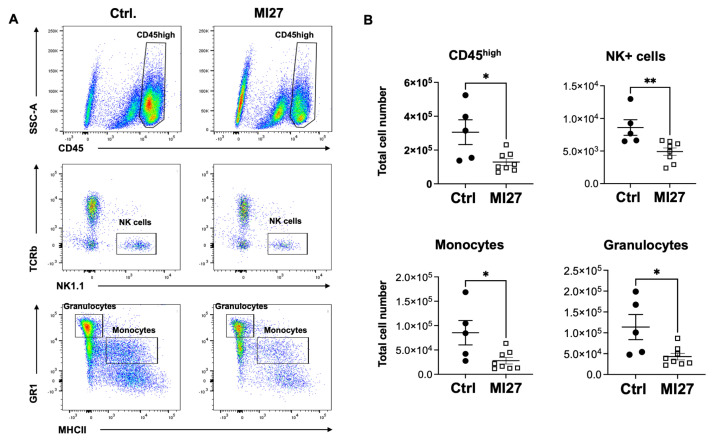
Intrathecal MI27 inhibited infiltration of immune cells to the CNS of mice with EAE. (**A**) Flow cytometric gating strategy to distinguish CD45^high^ leukocytes from CD45^dim^ microglia. Monocytes (CD45^high^CD11b^high^GR1^int^MHCII^+^), granulocytes (CD45^high^CD11b^high^GR1^high^MHCII^−^), and NK cells (CD45^high^CD11b^int^TCRb-NK1.1^+^) were gated from CD45^high^. (**B**) The total number of CD45^high^ cells was reduced in spinal cords from MI27 treated mice. Monocytes and granulocytes, as well as NK cells, were significantly reduced in the spinal cord tissues of mice upon intrathecal MI27 treatment (*n* = 5–8 per group). Data are presented as mean ± SEM. Results were analyzed using the non-parametric Mann-Whitney test. * *p* < 0.05, ** *p* < 0.01.

The results from histology suggested that the modulation of immune cell infiltration within the CNS can serve as a potential mechanism underlying the efficacy of MI27 treatment. To further characterize infiltrating immune cells in the CNS in response to intrathecal MI27, we conducted flow cytometry analysis in mice with EAE (Figure 3A). The results showed that the total number of CD45^high^ cells was significantly reduced in MI27-treated mice compared to control mice (Figure 3B). Further analysis of CD45^high^ cells demonstrated a decrease in the total numbers of infiltrating myeloid cell populations, including monocytes (CD45^high^CD11b^high^GR1^int^MHCII^+^) and granulocytes (CD45^high^CD11b^high^GR1^high^MHCII^−^). Additionally, the total number of NK cells (CD45^high^CD11b^int^TCRb^-^NK1.1^+^) was significantly lower (Figure 3B), whereas the number of T and B lymphocytes was not affected in MI27-treated mice.

### 2.4. Intrathecal MI27 Altered Inflammatory Programs in the CNS of Mice with EAE

**Figure 4:**
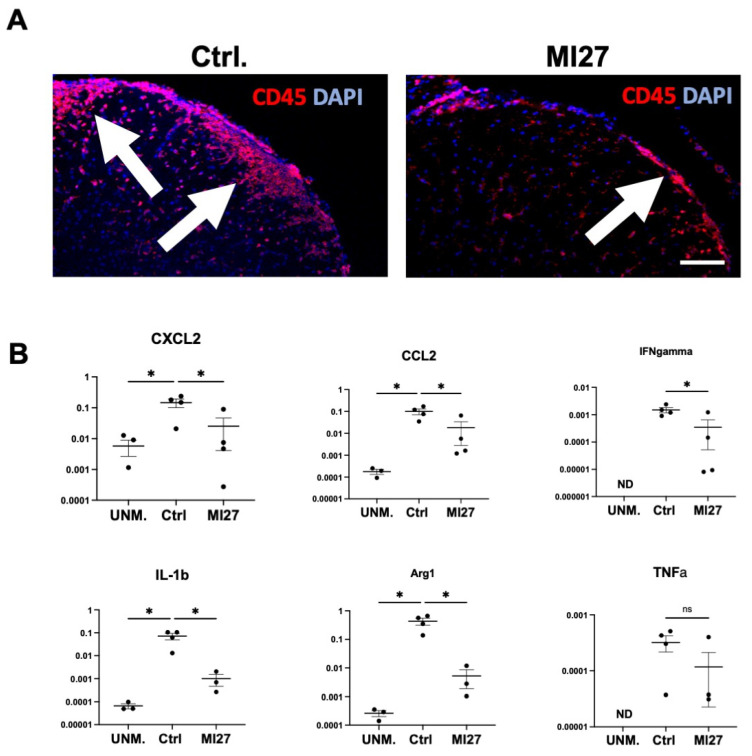
Intrathecal MI27 in mice with EAE-altered localization of CD45+ cells to the CNS. (**A**) Micrographs of spinal cord sections from mice with EAE that received MI27 or PBS intrathecally, stained with anti-CD45 antibody. Mice with EAE that were treated with intrathecal MI27 showed CD45+ cell accumulation in the meninges (arrow), whereas in control mice (ctrl), CD45+ cells were found in the spinal cord parenchyma (arrows). Scale bar: 100 µm. (**B**) RT-qPCR analysis of spinal cords showed chemokines, including *CCL2* and *CXCL2* as well as cytokines *IFNγ*, *IL-1β*, and *Arg1*, were significantly reduced upon intrathecal MI27 treatment at 1-day post-dose (*n* = 4 in each group). Unm = unmanipulated. ns = not significant. ND = not detected. Data are presented as mean ± SEM. Results were analyzed using the two-tailed Mann–Whitney u-test. * *p* < 0.05.

Flow cytometry results suggested that MI27 reduced the recruitment of CD45+ cells into the CNS. To further examine the distribution of CD45+ cells, we performed immunofluorescent staining on spinal cord sections from EAE mice intrathecally treated with PBS or MI27. At one day post-MI27 administration, CD45+ cells were primarily located in the leptomeningeal space (Figure 4A). In contrast, CD45+ cells were more abundant in the spinal cord parenchyma of control mice with EAE (Figure 4A).

To assess the impact of simultaneous activation of TLR7 and NOD2 downstream signaling on CNS inflammatory responses in mice with EAE, we analyzed the expression of inflammation-associated mediators using RT-qPCR. Intrathecal MI27 treatment resulted in significant downregulation of *CCL2* and *CXCL2* mRNA at one day post-dose (Figure 4B). Moreover, we found that treatment with intrathecal MI27 significantly reduced the levels of *IL-1β*, *IFN-γ*, and *Arg1* mRNA (Figure 4B). The levels of *CSF1*, *IFNα*, and *IL-6* and the levels of transcription factors including nuclear factor-kappa B (NF-κB), interferon regulatory factors 7 (*IRF7*) and *IRF3* were not affected, at one day post-treatment (Supplementary Figure S2).

## 3. Discussion

The present study highlights the potential therapeutic efficacy of synergistic targeting of innate receptors TLR7 and NOD2 in mitigating neuroinflammation and suppressing EAE progression. By harnessing the joint effects of these receptors, we induced robust anti-inflammatory responses characterized by the production of IFN I, which exerted protective effects in the context of EAE. MI27 treatment led to reduced recruitment of CD45 high cells, including monocytes, granulocytes, and NK cells, into the spinal cord. The reduced cellular infiltration was associated with the downregulation of chemokines including *CCL2*, and *CXCL2* as well as cytokines including *IL-1β*, and *IFNγ*, whereas MI27 did not affect the expression of *CSF1*, *CXCL10*, *IL-6*, *TNFα*, *IFNα*, *NF-κB*, *IRF3*, and *IRF7*.

Previous studies have shown that peripheral administration of imiquimod, a TLR7 ligand, to mice immunized for EAE improved symptoms by inducing IFN I production [13]. Conversely, research with TLR7 knockout mice indicated that TLR7 signaling exacerbates EAE [14]. In our previous study, we demonstrated that intrathecal imiquimod had minimal effects on IFN I induction, immune cell infiltration, and did not suppress EAE [4]. Similarly, stimulation of NOD2 has been found to promote EAE progression, with NOD2-deficient mice being resistant to EAE and displaying reduced numbers of T cells and activated dendritic cells in the CNS [15]. In the present study, we found that intrathecal administration of NOD2 ligand did not induce an optimal IFN I response and failed to suppress EAE. Interestingly, peripheral administration of MDP in EAE exhibited a significant beneficial effect both before and after disease onset [16]. Using NOD2 deficient mice, the study demonstrated that NOD2 signaling is crucial for MDP-dependent immune modulation and resistance to EAE [16]. Whether these differential effects of NOD2 stimulation is due to the timing of treatment or the rout of MDP administration needs further investigation. Interestingly, the combination of imiquimod and MDP resulted in robust IFN I production, suppression of EAE, and a reduction in the pro-inflammatory response. These results suggest that the distinct impact of intrathecally administered MI27 on EAE may be attributed to its synergistic effects, highlighting the importance of combined receptor stimulation for effective modulation of EAE progression.

Although several scenarios can be proposed, the molecular mechanisms behind this synergy remain poorly understood. One possible scenario is a direct interaction of signaling pathways downstream of NOD2 and TLR7. It is pertinent to consider the potential involvement of intracellular signaling pathways, such as NF-κB and IRFs, including IRF3 and IRF7, in mediating the anti-inflammatory effects of combined TLR7 and NOD2 activation. Both TLR7 and NOD2 signaling pathways converge on these transcription factors, which play critical roles in regulating immune responses and gene expression of inflammatory mediators, including IFN I production [17,18,19]. Although no changes in the levels of *IRF7*, *IRF3*, and *NF-κB* in response to MI27 were observed one day post-treatment, we cannot exclude the possibility that the synergistic activation of TLR7 and NOD2 leads to activation of these pathways at earlier time points. This is plausible, as we observed strong but transient IFN I induction at 4 h that contributed to the observed IFN I-dependent suppression of neuroinflammation. Given the intricate crosstalk between TLR7, NOD2, NF-κB, and IRF signaling pathways, it is plausible that the therapeutic effects of combined receptor activation observed in our study may involve complex interactions at the level of intracellular signaling cascades. Future investigations into the specific roles of NF-κB and IRFs in mediating the anti-inflammatory and immunomodulatory effects of synergistic receptor targeting will provide valuable mechanistic insights and may identify additional therapeutic targets for MS and related autoimmune disorders.

It is unclear whether the therapeutic effect of MI27 is achieved through the simultaneous activation of NOD2 and TLR7 on a single cell or whether these two ligands act separately, targeting different cells. It is therefore important to study the cellular target of MI27. This would require the usage of covalently conjugated NOD2/TLR7 agonists or fluorescent-labeled TLR7- and NOD2- agonists [7,8,20].

The reduction in cytokines, including *IL-1β* and *IFNγ*, and chemokines, including *CCL2* and *CXCL2* in mice with EAE treated with MI27, may be attributed to two potential mechanisms. First, MI27 may decrease the infiltration of immune cells into the CNS tissue. This might be due to the robust induction of IFN I by MI27, which has been demonstrated to decrease immune cell infiltration into the CNS as well as reduction of *IFNγ* and *FoxP3* expression in mice with EAE [21], which was also downregulated in our study. Second, MI27 may induce an anti-inflammatory state by preventing the induction of proinflammatory mediators within the CNS. The current study suggests that MI27 blocks the infiltration of proinflammatory/encephalitogenic immune cells and reduces proinflammatory responses. In this regard, monocytes, granulocytes, and NK cells are among the cells that were significantly reduced in the CNS of mice treated with MI27, consistent with the chemokine and cytokine profile. Monocytes and granulocytes play a critical role in MS-like disease, and their recruitment to the CNS is essential for EAE progression [22,23]. Our findings are in agreement with these studies and suggest that MI27 reduces infiltrating monocytes and granulocytes, thereby preventing EAE progression. NK cells are a specialized population of innate lymphoid cells that help control local immune responses. Several studies demonstrated that the NK cells play a detrimental role in EAE, and the presence of NK cells, and more specifically NK cell-derived IFNγ, are critical for the development of EAE pathology [24,25,26]. Our findings support these notions. However, others have suggested a protective role for NK cells [27]. Future studies may clarify which NK cells subtypes are involved in MI27-mediated response.

In conclusion, our study demonstrates that simultaneous targeting of innate receptors TLR7 and NOD2 leads to synergistic induction of IFN I and effectively suppresses EAE progression in a murine model of MS. These findings support the therapeutic potential of combinatorial approaches aimed at modulating neuroinflammation and restoring CNS homeostasis in MS. Future studies focusing on elucidating the precise mechanisms underlying the synergistic effects of TLR7 and NOD2 signaling pathways will facilitate the development of novel treatment strategies for MS and related autoimmune disorders.

## 4. Materials and Methods

### 4.1. Mice

Female albino IFNβ+/Δβ-luc mice (IFNβ/luciferase reporter mice) [12] and IFNAR1-KO mice (C57BL/6 background) were specifically bred and maintained under standardized conditions at the Biomedical Laboratory, University of Denmark. Female C57BL/6j mice were purchased from Taconic Europe A/S (Lille Skensved, Denmark). All procedures involving animals were conducted in strict accordance with the guidelines provided by the Danish Animal Experiments Inspectorate, and the study was conducted under the approved protocol number 2020-15-0201-00652.

### 4.2. EAE Induction

Experimental autoimmune encephalomyelitis (EAE) was induced in both C57BL/6 and IFNAR1-KO mice following established protocols [2]. Briefly, mice were immunized with a 100 µL emulsion containing 100 µg of myelin oligodendrocyte glycoprotein (MOG)p35-55 (TAG Copenhagen A/S, Copenhagen, Denmark) in complete Freund’s adjuvant (BD Biosciences, Lyngby, Denmark), supplemented with 200 µg of heat-inactivated Mycobacterium tuberculosis (BD Biosciences), administered subcutaneously into each hind flank. Furthermore, mice received an intraperitoneal injection of Bordetella pertussis toxin (0.3 μg, Sigma-Aldrich, Søborg, Denmark) at the time of immunization and again 1 day post-immunization. To monitor disease progression, mice were weighed daily and evaluated for EAE symptoms, graded on a scale ranging from 0 to 5 based on predefined criteria. The EAE grades were defined as follows: grade 0, no signs of disease; grade 1, weak or hooked tail; grade 2, floppy tail, indicating complete loss of tonus; grade 3, floppy tail and hind limb paresis, grade 4: floppy tail and unilateral hind limb paralysis; grade 5, floppy tail and bilateral hind limb paralysis. Due to ethical reasons, mice were sacrificed when they reached grade 5 or if hind limb paralysis persisted for 2 days.

### 4.3. Intrathecal Injection

Intrathecal injections were performed under anesthesia induced by inhalation of isoflurane (Abbott Laboratories, Chicago, IL, USA). A 30-gauge needle, bent at a 55° angle with a 2 mm tip, attached to a 50 μL Hamilton syringe, was used for precise delivery into the intrathecal space. Mice received injections of either 12.5 µg imiquimod (R837, Invivogen, San Diego, CA, USA), 25 µg Muramyl dipeptide (MDP, L-D isomer, active, Invivogen), 25 µg MDP control (L-L isomer, inactive, Invivogen) + imiquimod (12.5 µg), MI27 (25 µg MDP + 12.5 µg imiquimod), or phosphate-buffered saline (PBS). These doses were selected based on preliminary experiments demonstrating optimal effects and were consistently used throughout the study.

### 4.4. In Vivo Imaging

To visualize and quantify luciferase activity, in vivo imaging was performed according to established protocols. IFN-β+/Δβ-luc mice were injected intraperitoneally with D-luciferin (150 mg/kg) and subsequently monitored at 4-, 24-, and 48 h post dose, using an IVIS 200 imaging system (CaliperLS) (DaMBIC. Photon flux emitted from luciferase activity was quantified using Living Image 4.4 software (CaliperLS).

### 4.5. Tissue Processing

Upon completion of experiments, mice were euthanized using an overdose of sodium pentobarbital (100 mg/kg, Glostrup Apotek, Glostrup, Denmark) and subsequently perfused with ice-cold PBS. For subsequent analysis, CNS tissue was either placed in ice-cold PBS for flow cytometry or post-fixed with 4% paraformaldehyde (PFA), followed by immersion in 30% sucrose in PBS for histological processing. Tissue sections (16 μm thick) were obtained using a cryostat (Leica). Additionally, CNS tissues, intended for reverse transcriptase–quantitative polymerase chain reaction (RT-qPCR) analysis, were stored in Trizol Reagent (Ambion, Austin, TX, USA) at −80 °C until RNA extraction.

### 4.6. Flow Cytometry

A single-cell suspension was obtained by forcing the CNS tissue through a 70 μm cell strainer (Falcon, Miami, FL, USA) with Hank’s buffered salt solution (HBSS, Gibco, Waltham, MA, USA) supplemented with 2% fetal bovine serum (FBS, Merck, Germany). Myelin was cleared by resuspending cells in 37% Percoll (GE Healthcare Bio-sciences AB, Chicago, IL, USA) in a buffer consisting of PBS pH 7.2. The myelin layer was removed, and the cell pellet was washed and incubated in a blocking solution containing HBSS, 2% FBS, anti-CD16/32 antibody (1 µg/mL, Clone 2.4G2, BD Biosciences, San Jose, CA, USA), and Syrian hamster IgG (50 μg/mL, Jackson ImmunoResearch Laboratories Inc., West Grove, PA, USA). The cells were then labeled with fluorophore-conjugated antibodies (BioLegend, San Diego, CA, USA), including anti-CD45 (clone 30-F11), CD11b (M1/70), GR-1 (RB6-8C5), NK1.1 (PK136), and TCRβ (H57-597), MHC II (M5/114.15.2). Fluorescence was measured using an LSRII flow cytometer (BD Biosciences) with FACS Diva^TM^ software version 6.1.2 (BD Biosciences) and analyzed with Flow logic (Inivai Technologies, Victoria, Australia) and FlowJo (Becton, Dickinson & Company, Franklin Lakes, NJ, USA) as described previously [2,28].

### 4.7. Histology

Luxol Fast Blue (LFB) and cresyl violet staining were performed as described [2]. To investigate glial response, frozen spinal cord sections from C57BL/6 mice with EAE, intrathecally injected with MI27 or PBS respectively, were stained with anti-IBA1- or CY3-conjugated anti-glial fibrillary acidic protein (GFAP, C9205-2ML, Sigma, St. Louis, MO, USA), anti-MOG antibody, anti-MBP antibody and PE- conjugated anti-CD45 antibody as described previously [2,4,28]. Images were acquired using an Olympus DP71 digital camera mounted on an Olympus BX51 microscope (Olympus, Ballerup, Denmark). Images were combined using Adobe Photoshop CS3 (Adobe Systems Denmark A/S, København, Denmark).

### 4.8. RNA Isolation and RT-qPCR

RNA extraction from spinal cords was performed using TRIzol reagent in accordance with the manufacturer’s protocol. RNA was converted into cDNA using a high-capacity cDNA reverse transcription kit (Applied Biosystems, Waltham, MA, USA). RT-qPCR was performed using an ABI Prism 7300 sequence detection system (Applied Biosystems), using primers and probes described earlier [2,4,28] Ct values were determined, and the results presented as genes of interest relative to 18sRNA (2^ΔCT^ method).

### 4.9. Statistical Analysis

Outliers were identified and excluded using the ROUT method prior to further statistical analysis. Comparisons between two groups were conducted using the non-parametric Mann–Whitney test. For comparisons among three groups, one-way ANOVA followed by a multiple comparison test was utilized. For analyzing IFNβ induction in IFNβ/luciferase reporter mice, a paired Student’s *t*-test was performed. All statistical analysis was performed using GraphPad Prism version 10 (Graphpad Software Inc., La Jolla, CA, USA). Results are presented as means ± SEM. Values of *p* < 0.05 were considered significant.

## Data Availability

The data will be available upon request.

## Supplementary Materials

The following supporting information can be downloaded at: https://www.mdpi.com/article/10.3390/ijms25137462/s1.
