# Supplementary material for: Synergistic Targeting of Innate Receptors TLR7 and NOD2 for Therapeutic Intervention in Multiple Sclerosis
Source: Int J Mol Sci. 2024 Jul 7;25(13):7462. doi: 10.3390/ijms25137462 (PMC11242768; doi:10.3390/ijms25137462)
Supplement: Supplementary file 1 [file ijms-25-07462-s001.zip › ijms-3072705-supplementary.pdf]

International Journal of
Molecular Sciences

Supplementary Materials:

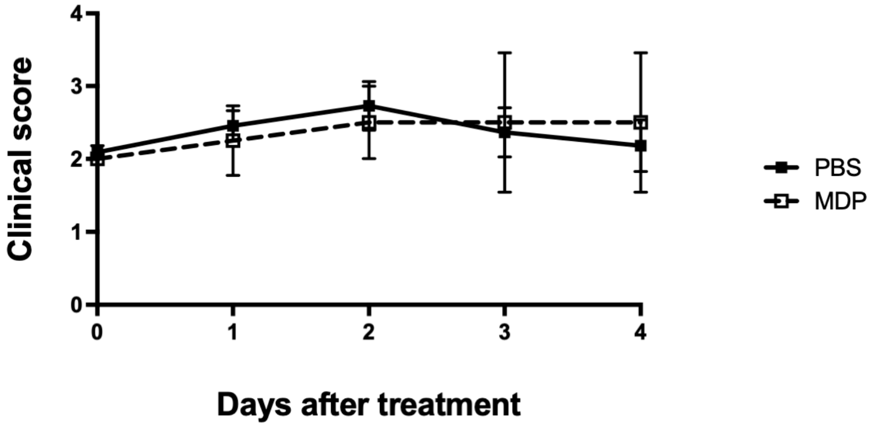

Supplementary Figure S1. Intrathecal MDP had no effect on EAE progression. A) C57BL/6 mice were immunized with MOGp35-55 to induce EAE, and at disease onset, they received intrathecal MDP or PBS, and clinical signs were scored daily. Intrathecal MDP showed no effect on disease compared to control mice (n=4-11 in each group).

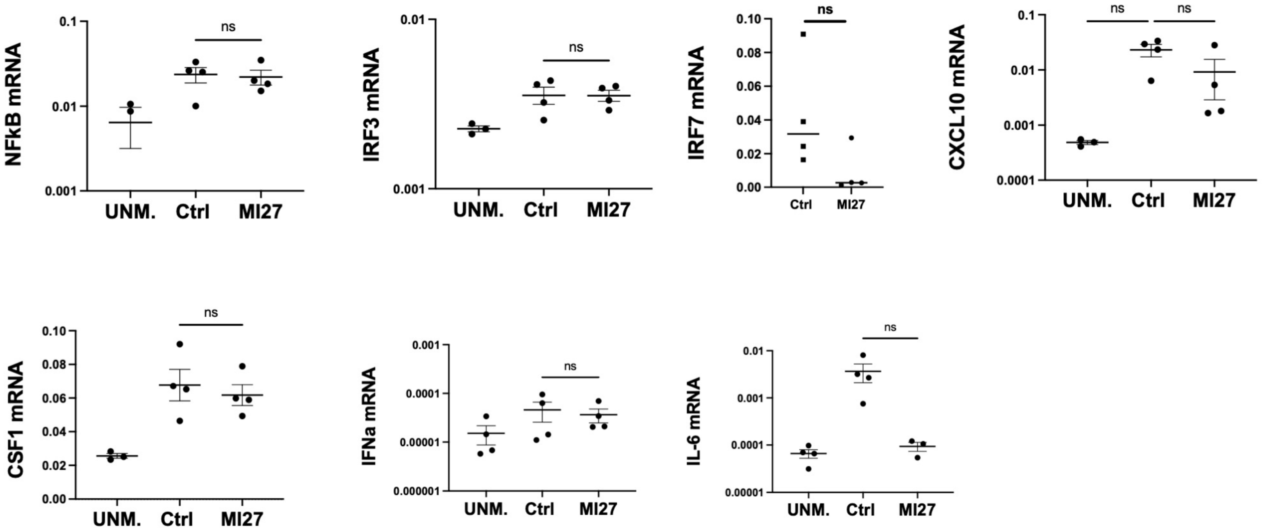

Supplementary Figure S2. RT-qPCR analysis of spinal cords showed that intrathecal MI27 had no effect on the levels of CSF1, IL-6, IFNα, NF-κB, IRF3, CXCL10, or IRF7 at 1 day post-dose (n=4 in each group). Unm = unmanipulated. Data are presented as mean ± SEM. Results were analyzed using the two-tailed Mann–Whitney Utest.
